# Nitrogen-doped carbon paper with 3D porous structure as a flexible free-standing anode for lithium-ion batteries

**DOI:** 10.1038/s41598-017-07345-y

**Published:** 2017-08-10

**Authors:** Hua Zhang, Juntan Yang, Haoqing Hou, Shuiliang Chen, Haimin Yao

**Affiliations:** 1 Department of Chemistry and Chemical Engineering, Jiangxi Normal University, Nanchang, 330022 China; 2 Department of Mechanical Engineering, The Hong Kong Polytechnic University, Hung Hom, Kowloon, Hong Kong SAR China

## Abstract

In this paper, a novel nitrogen-doped carbon paper (NCP) with both highly dense three-dimensional cellular structure and excellent bending flexibility is fabricated by pyrolyzing a melamine foam under compression. When serving as a free-standing anode for lithium-ion batteries, the NCP electrode delivers a reversible capacity up to 329.8 mA h g^−1^ after 200 cycles at 0.5 A g^−1^ (1.34 C) and 126.5 mA h g^−1^ after 500 cycles at 8.0 A g^−1^ (21.5 C). Such electrochemical performance is much higher than those of the counterparts prepared by pyrolysis without compression and can be mainly attributed to (a) the 3D highly dense interconnected carbon network with numerous junctions which can facilitate the efficient electron transfer and provide short transportation paths for lithium ions; and (b) the excellent mechanical flexibility and self-standing capability which exempt the use of binder, conductive additive and current collector. The NCP electrode implies a great promise of application in the high-performance Li-ion batteries for the flexible and wearable electronics.

## Introduction

Rechargeable lithium-ion batteries (LIBs), as the main power supplies, have gained great attention because of their high energy density, high output voltage, and environmental friendliness^1^. A conventional procedure to prepare LIB anode starts from the produce of a slurry by mixing electroactive powder (*e.g*. graphite and Si particles) with conductive carbon additives and a polymeric binder dissolved in a solvent like water. The slurry then is spread onto a current collector (*e.g*. copper foil) and dried. The electrodes prepared by the conventional protocol have several drawbacks. First, the metallic current collectors, conductive additives, and binders are electrochemically inactive and do not contribute to the lithium storage, thus greatly reduce the overall energy and power densities of LIBs^2^; Secondly, the use of polymeric binders greatly reduces the electrical conductivity and prevents the access of ions to the surface of the active materials. Additionally, the mass loading of active materials is relatively low because high mass loading causes delamination and microcracks easily. Moreover, such composite anode material is fragile and therefore is not applicable to the flexible devices and wearable electronics. Development of free-standing electrode materials with light weight, high electrical conductivity, and high mechanical flexibility is in great demand.

Flexible carbon materials, as free-standing electrodes in LIBs, have attracted numerous attentions for their tunable structures, high chemical stability, and good electrical conductivity^3^. A variety of flexible carbon-based materials with different forms and structures, such as carbon nanotube (CNT) film/sponge^4–10^, graphene paper/foam^11–19^ and CNT/graphene composite papers^20^, have been developed as free-standing electrodes by diverse approaches. Although the good performance exhibited by these flexible carbon-based materials implies their great promise of application in high-performance LIBs, many practical challenges still exist. For instance, the aligned carbon nanotube (CNT) and graphene film prepared by chemical vapor deposition deliver high areal capacity^9, 18^. However, the complex preparation processes and high manufacturing cost impede their wide application in engineering. As the most commonly used fabrication method for graphene and CNT-based composite or hybrid flexible electrodes, vacuum ultrafiltration still suffers from the restacking of graphene sheets and the residual impurity components (*e.g*., surfactants), which give rise to high irreversible capacity and fast capacity fading (especially at high current density)^2^. Recently, electrospinning has also been applied as an alternative fabrication technique for the free-standing carbon-based electrodes^21–24^. But this technique also has some shortcomings such as slow electrode preparation rates, residual impurity components and overmuch pore volume in the electrospun materials. New fabrication technology with scaling-up capacity is needed for developing carbon-based electrode materials with high flexibility and excellent electrochemical performance.

In our earlier work^25, 26^, an elastic nitrogen-doped carbon foam (ECF) was fabricated simply by direct pyrolysis of melamine foam (MF). The obtained carbon foam displayed a high specific surface area, a low density (5 mg cm^−3^) and a high electrically conductivity. These properties make ECF a promising candidate for the free-standing flexible anode material for high-performance LIBs. Recently, a few similar works also reported the developments of mesoporous carbon foams as anodes for LIBs^27^. However, the capacity (*e.g*., ~180 mA h g^−1^ at 200 mA g^−1^) and cycling stability of the LIBs with the as-synthesized carbon foams used as the electrodes is still unsatisfactory, which is probably due to the low density of the carbon foams. In this paper, we aim to improve the technique by imposing constant compression on the MF during pyrolysis. A highly dense and flexible nitrogen-doped carbon paper (NCP) is obtained and found to possess a higher gravimetric capacity, cycling performance, and rate performance in comparison to those of the counterparts prepared without compression.

## Results

### Structure and physical properties of NCPs

**Figure 1:**
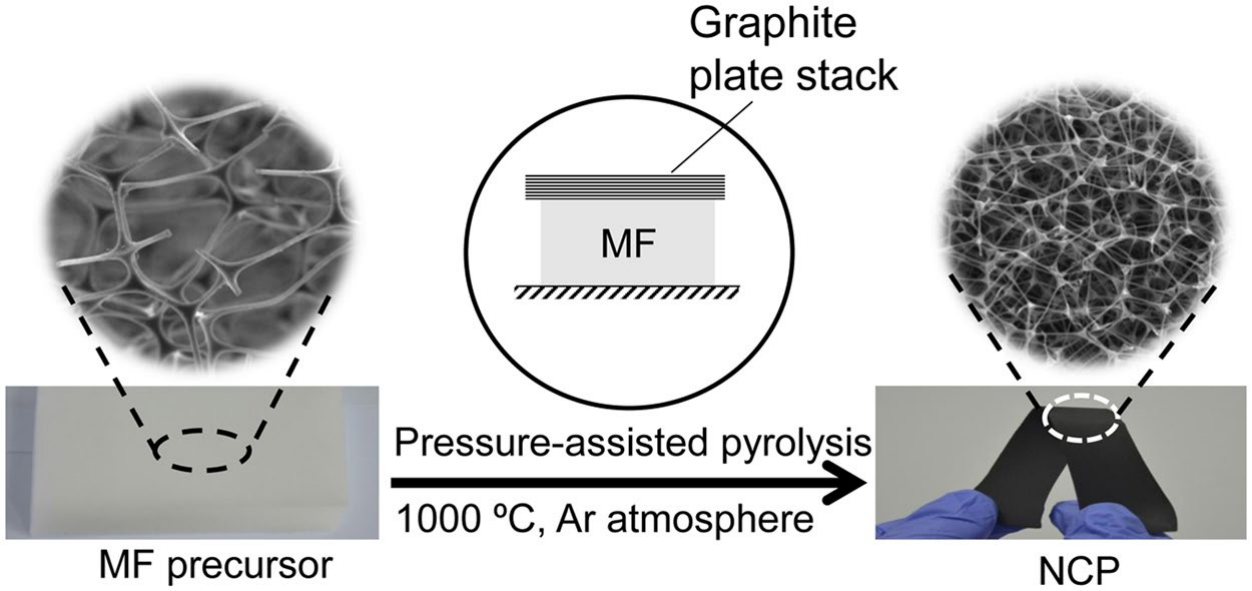
Schematic of the fabrication process of NCPs.

As illustrated in Fig. 1, the highly dense and flexible NCP is fabricated simply by a pressure-assisted pyrolysis method. The thickness of the NCP depends on the pressure applied. The higher the pressure, the thinner the obtained NCP. In our experiments, pressures of 200, 400, 600 kPa were applied, resulting in NCPs with thicknesses around 800, 600 and 200 μm, respectively. An even higher pressure is tried and found to cause pulverization of the NCP. According to the pressure under which the pyrolysis is performed, the obtained NCP samples are designated as NCP-200k, NCP-400k, and NCP-600k, respectively. For comparison, control samples are also prepared under the same synthesis conditions but without applying any pressure. The obtained samples are low-density elastic carbon foams of about 6 mm thick and designated as NCP-0.

**Figure 2:**
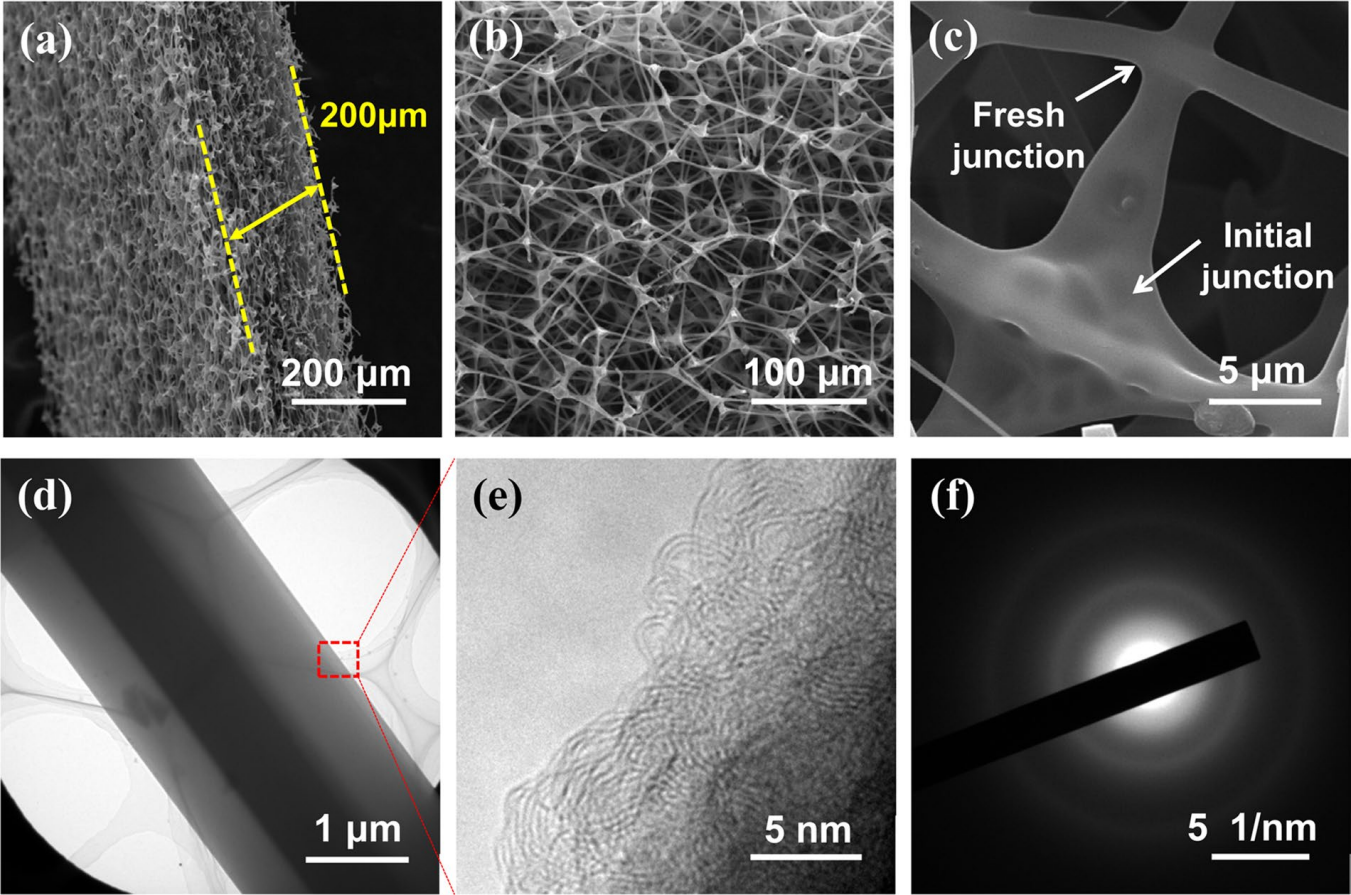
Ultramicroscopic structures of the obtained NCP-600k. SEM images of (**a**) cross section and (**b**, **c**) the surface subjected to compression during fabrication; (**d**,**e**) high-resolution TEM image of a fiber and (**f**) the corresponding SAED pattern.

Without exception, all the NCPs, no matter fabricated under compression or not, exhibit three-dimensional cellular structure with cells constructed by microscopic prismatic fibers (Fig. 2 and Figure S2). Such cellular structure is certainly inherited from their precursor − MF. Figure 2 shows the SEM and TEM images of the as-prepared NCP-600k. From Fig. 2a, it can be seen that the thickness of NCP-600k is about 200 μm, which is 1% of the original thickness of MF. It is interesting and worthwhile to note that a large number of fresh junctions between the carbon fibers are formed during the pressurized pyrolysis in the NCP-600k sample, as shown in Fig. 2c. The formation mechanism of these fresh junctions is proposed as follows. Upon compression, more MF fibers will intersect with the others. During pyrolysis, tar oil forms and deposits on the fibers especially at the inter-fiber intersections. At high temperature, the tar oil is pyrolyzed into carbon. The intersecting fibers then are welded together by the carbon and new junctions are formed. The high-resolution TEM (HRTEM) image (Fig. 2e) indicates that carbon fibers of the NCP-600k mainly consist of amorphous carbon structure. The corresponding selected area electron diffraction (SAED) pattern (Fig. 2f) exhibits the typical characteristics of amorphous materials. As expected, the porous structures of the NCPs exhibit dependence on the pressure applied during the pyrolysis, as shown in Figure S2.

**Figure 3:**
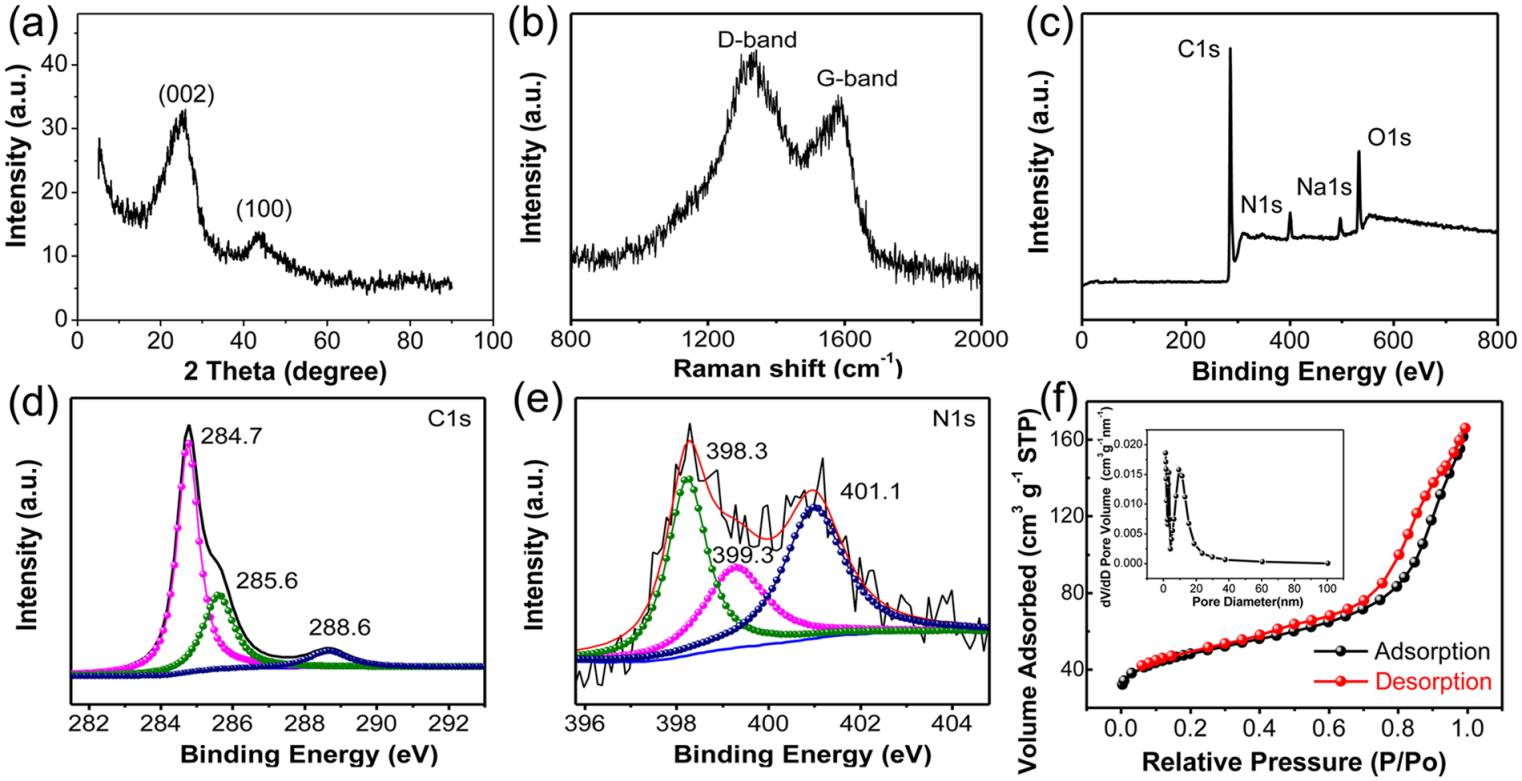
Characteristic spectra of NCP-600k. (**a**) XRD pattern; (**b**) Raman spectrum; (c-e) XPS spectra, (**c**) survey spectrum, (**d**,**e**) are high-resolution spectra of the C1s and N1s, respectively; (**f**) N_2_ adsorption/desorption isotherm curves with inset being the pore-size distribution curve.

Figure 3a shows the X-ray diffraction (XRD) pattern obtained from a sample of NCP-600k. The two peaks corresponding to the (002) and (100) reflections imply the existence of graphitic carbon. However, the relative wide breadth of both peaks implies the low degree of graphitization. Raman spectrum in Fig. 3b exhibits two peaks at ~1329 and ~1584 cm^−1^, corresponding to the shifted D-band and G-band. The intensity ratio between D band and G band (*I*_*D*_/*I*_*G*_), is estimated to be 1.14, indicating a signature of low-crystallinity carbon with disordered orientations, which is consistent with the HRTEM image and the XRD pattern. Such turbostratic carbon structure is believed beneficial for the reversible lithium ion storage^23^. X-ray photoelectron spectroscopy (XPS) survey spectrum in Fig. 3c shows that the NCP mainly contains C, O and N elements. The high-resolution C1s spectrum (Fig. 3d) displays three peaks at 284.7, 285.6 and 288.6 eV, corresponding to the carbons in C–C bond, C–N bond and C = O bond, respectively^28^. Three types of N species, pyridinic (N-6, 398.3 eV), pyrrolic-N (N-5, 399.3 eV) and quaternary (N-Q, 401.1 eV) nitrogen, are identified in the carbon fibers, as shown by the high-resolution N1s spectrum (Fig. 3e). As reported in the literature^29, 30^, N-6 nitrogen and N-Q nitrogen are both sp^2^ hybridized species which can significantly improve the electrochemical capability of carbon materials by tailoring the electronic and chemical properties of the carbon host. Moreover, since the N-6 nitrogen mainly results from the substitution of nitrogen atoms for the carbon atoms on the edges or defect sites of the graphitic carbon layer, N-6 nitrogen holds a low energy barrier for lithium penetration and can serve as electrochemically active sites for enhancing the lithium storage capacity and rate capability^31^. Using XPS analysis, the content of nitrogen doping in NCP-600k is determined to be around 5.24%, in which 39.04% is in N-6 nitrogen, 36.16% is in N-5 nitrogen and 24.8% is in N-Q nitrogen. Compared to those reported in the previous work^30, 32^, the content of nitrogen doping, especially that of the N-6 nitrogen, in our NCP-600k is higher and is believed to benefit the electrical conductivity and provide additional anchoring sites for Li-ions, giving rise to higher lithium storage capacity^30^.

In addition to the microscopic cellular structure as shown in Fig. 2, the NCPs also exhibit porous structure at the nanoscale. The nitrogen absorption/desorption isotherms in Fig. 3f indicate that the microscopic carbon fibers are not solid. Instead, they contain numerous mesopores with the dominant diameter being around 10 nm. These mesopores are believed to result from the thermal decomposition of MF into a large amount of gas including CO, CO_2_, and NH_3_^25^. Such hierarchical porous structure endows the NCPs with the high specific surface (see Table 1), which is found to depend on the applied pressure during pyrolysis. The higher the pressure, the lower the specific surface area. Among our samples, NCP-600k exhibits the lowest specific surface area of 86.7 m^2^ g^−1^. Such pressure-dependence of the specific surface area is probably due to the stagnant argon flow in the pyrolyzed MF under compression. During pyrolysis, volatile substances released from MF cannot be vented easily from a pressurized MF. The trapped substances will deposit on the MF fibers, forming tar oil which will be converted into carbon deposition and fill in the mesopores. In comparison to the specific surface area, the density of NCPs exhibits even higher dependence on the pressure. The densest sample, NCP-600k, possesses a density of around 114 mg cm^−3^ which is more than 16 times of that of NCP-0.

**Table 1:** Particulars of the synthesized NCP samples.

Sample	Dimensions L × W × H (cm)	Mass (mg)	Porosity (%)	Density (mg cm^−3^)	Specific surface area (m^2^ g^−1^)	Bulk conductivity (S cm^−1^)	Critical Curvature (mm^−1^)	Flexural modulus (MPa)
NCP-0	2 × 1.5 × 0.6	12.3	99.62	6.84	170	6 × 10^−2^	0.12	0.33
NCP-200k	2 × 1.9 × 0.08	22.5	95.89	73.98	159	0.33	0.15	9.78
NCP-400k	1.9 × 1.8 × 0.06	18.5	94.99	90.18	138	1	0.23	27.13
NCP-600k	2.5 × 2 × 0.02	11.4	93.67	113.94	86.7	2.1	0.74	38.5

As structure plays an important role in determining the properties of materials, the preceding structural differences in NCPs, which are caused by the applied pressure during pyrolysis, directly lead to a discrepancy in their properties. For example, the NCP-600k exhibits an electrical conductivity of 2.1 S cm^−1^, which is 35 times higher than that of the NCP-0 (6 × 10^−2^ S cm^−1^) sample. Besides the high density, another reason accounting for such high electrical conductivity of NCP-600k is the numerous inter-fiber junctions formed during the pressurized pyrolysis, as shown in Fig. 2c.

**Figure 4:**
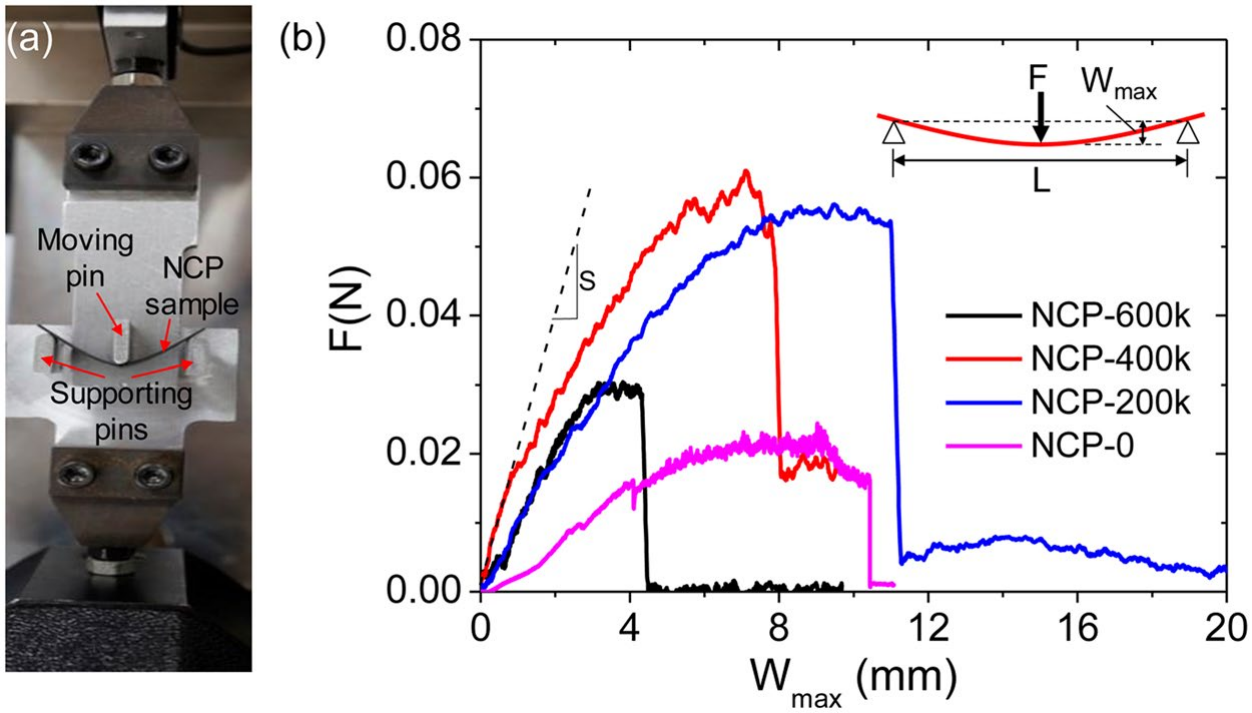
(**a**) Setup of 3-point bending test and (**b**) the obtained force-deflection curves of different NCP samples. The maximum bearable curvature and the flexural modulus of NCPs are deduced from ${\kappa }_{c}=12{W}_{{\rm{\max }}}^{c}/{L}^{2}$ and *E*_f_ = *L* ^3^ *S*/4*ah* ^3^ respectively, where *L* is the span between two supporting pins, ${W}_{\max }^{c}$ stands for the deflection of the sample at the middle point when fracture, *a* and *h* are the width and thickness of the sample’s cross section respectively, and *S* is the slope of the initial portion of the force-deflection curve, as indicated for the curve of NCP-400k as an example.

In addition to electrical conductivity, NCPs also exhibit excellent flexibility, which can be seen from the huge bearable deformation under bending. Such flexibility can be quantified by the maximum bearable curvature before fracture (${\kappa }_{c}$) under 3-point bending test (Fig. 4a). Among our NCP samples, NCP-600k exhibits the highest ${\kappa }_{c}$ of 0.47 mm^−1^. Meanwhile, all the NCPs exhibit relative lower stiffness compared to the traditional carbon-based anode materials. According to the force-deflection curves obtained from the 3-point bending experiments (Fig. 4b), the flexural modulus of each NCP sample can be deduced through *E*_f_ = *L* ^3^ *S*/4*ah* ^3^ and is found in the range of 0.33–38.5 MPa, depending on the applied pressure during pyrolysis. The particulars of all NCP examples are summarized in Table 1.

### Electrochemical performance of the NCPs

**Figure 5:**
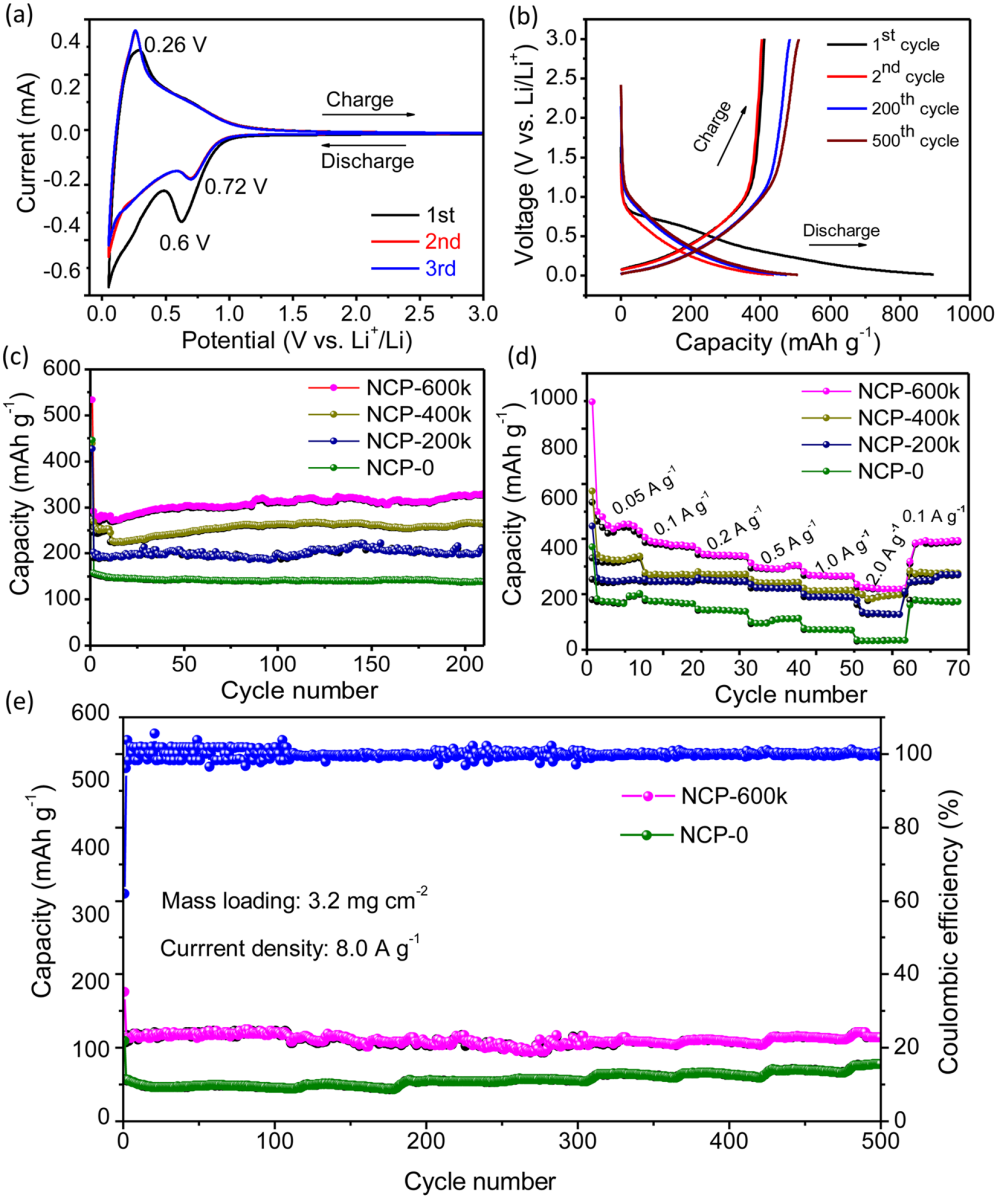
(**a**) Cyclic voltammograms of NCP-600k electrode for the initial three cycles at a scan rate of 0.1 mV s^−1^ in the voltage range of 0.01–3.0 V; (**b**) Galvanostatic charge-discharge voltage profiles of the NCP-600k electrode at a current density of 0.05 A g^−1^; (**c**) Cycling performance of the NCP electrodes at a current density of 0.5 A g^−1^; (**d**) Rate capability of the NCP electrodes at different current densities; (**e**) Cycling performance of the NCP-0 and NCP-600k electrodes at a current density of 8.0 A g^−1^. For clarity, only the efficiency of NCP-600k is shown. The coulombic efficiency of NCP-0 is similar and close to 100% as well.

The electrochemical performance of the obtained NCPs was evaluated in a coin half-cell without using any additive or metallic current collector. Figure 5a shows the CV curves in the first three cycles of the NCP-600k. It can be seen that a distinct cathodic peak occurs at ~0.60 V, which indicates the decomposition of the organic electrolyte accompanied by the formation of solid electrolyte interphase (SEI) films on the electrode-electrolyte interface^8^. This peak is stabilized at a higher potential of ~0.72 V with a weaken peak intensity in the following cycles, indicating that stable SEI films were almost formed during the first cycle. In the anodic scans, a broad peak at 0.26 V is observed, which could be ascribed to the extraction of lithium from carbon^33^. Besides, the peak positions and intensities after the second cycle almost overlap, implying the good reversibility of the electrode.

Figure 5b shows the charge-discharge profiles of the NCP-600k electrode in the 1^st^, 2^nd^ 200^th^ and 500^th^ cycles at a current density of 50 mA g^−1^. The discharge curve of the 1^st^ cycle exhibits a potential plateau below ~0.75 V, which can be attributed to the irreversible electrolyte decomposition and formation of the SEI layer. The NCP-600k electrode displays an initial discharge capacity of 895 mA h g^−1^ and a reversible capacity of 480 mA h g^−1^, giving rise to the initial coulombic efficiency of 53.8%. Interestingly, the reversible capacity of NCP-600k decreases from ~438 mA h g^−1^ at the 2^nd^ cycle to ~428 mA h g^−1^ at the 5^th^ cycle, but then increases to ~483 mA h g^−1^ at the 200^th^ cycle and maintains at 509 mA h g^−1^ up to 500 cycles. Compared with the theoretical capacity of graphite (372 mA h g^−1^), such high reversible capacity can be attributed to the cooperation of hierarchical porous structure and nitrogen doping in the NCPs^13, 34^. It should be noted that the reversible capacity and initial coulombic efficiency of the NCPs decreases with the applied pressure during pyrolysis. In contrast, the NCP-0 exhibits reversible capacity of 280 mA h g^−1^ (Figure S3) and initial coulombic efficiency of 30.4% only. Such low initial coulombic efficiency can be mainly attributed to the formation of SEI layer on the electrode–electrolyte interface or the irreversible insertion of lithium ions into carbon. The larger the specific surface area, the lower the initial coulombic efficiency.

Figure 5c shows the cycle performance of the NCPs electrodes at a current density of 0.5 A g^−1^. For the purpose of activation, the first ten cycles are operated at a low current density of 0.2 A g^−1^. It can be noticed that the capacity of the NCP-600k electrode progressively increases after a few (~11) cycles from 280 mA h g^−1^ to 329.8 mA h g^−1^. A discharge capacity of 329.8 mA h g^−1^ and charge capacity of 328.7 mA h g^−1^ are maintained after 200 cycles with coulombic efficiency being around 100%. Such remarkable increasement of the capacity may be attributed to the capability of carbon fiber surface of absorbing Li ions as reported in the previous work^24^. A similar increasement of capacity is also observed in the electrodes of NCP-200k and NCP-400k. In contrast, the capacity of the NCP-0 electrode gradually reduced from the initial value of 157.4 mA h g^−1^ to 137.9 mA h g^−1^ after 200 cycles, which might be due to the relatively higher electronic resistance and longer electronic transport paths.

Figure 5d shows the rate performance of the NCP electrodes at various current densities. Obviously, the rate performance of the NCP-600k electrode is superior compared to those of the other samples. The NCP-600k electrode delivers capacities of 492.1, 415.8, 375.5, 320, 293.5 and 240 mA h g^−1^ at current densities of 0.05, 0.1, 0.2, 0.5, 1.0 and 2.0 A g^−1^, respectively. It should be noted that even at a high current density of 2.0 A g^−1^, ~57.8% of the capacity at 0.1 A g^−1^ can still be achieved, which is much higher than that of the NCP-0. Furthermore, the capacity of NCP-600k recovers to 428 mA h g^−1^ when the current density is reduced back to 0.1 A g^−1^, indicating the excellent reversibility of the NCP-600k electrode. The cycling performance of the NCP electrodes at an even higher current density of 8.0 A g^−1^ is also investigated and the results are shown in Fig. 5e. Even after 500 cycles, the capacity of the NCP-600k electrode is still kept at 126.8 mA h g^−1^ with coulombic efficiency being around ~100%, showing the excellent rate capability and high stability. In contrast, the capacity of the NCP-0 electrode delivers capacity of 60 mA h g^−1^ only. To the best of our knowledge, our NCP electrode exhibits much better rate performance in comparison to most of the existing flexible, free-standing metal-free anode materials, as shown in Table S1.

**Figure 6:**
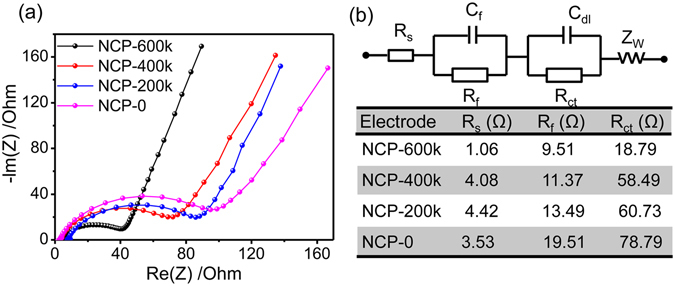
(**a**) EIS of the NCP electrodes in the frequency range of 100 kHz to 0.01 Hz; (**b**) Equivalent circuit and corresponding parameters obtained from Nyquist plots.

In order to understand the superior rate performance and cycling capability of NCP electrodes, electrochemical impedance spectroscopy (EIS) are conducted. The obtained Nyquist plots and the fitting parameters of the equivalent circuit model are shown in Fig. 6. In the equivalent circuit, *R*_s_ represents the solution resistance. *R*_f_ and *C*_f_ stand for the resistance and capacitance corresponding to Li-ions migration through the surface film, respectively. *R*_ct_ is the charge-transfer resistance related to the electrical conductivity of electrodes and the transport of Li-ions in the surface film/solution interface, and *C*_dl_ reflects the interfacial capacitance. And *Z*_w_ is the Warburg resistance related to Li-ions diffusion process^35, 36^. The corresponding impedance parameters extracted from the Nyquist plots are listed in Fig. 6b. Obviously, the charge-transfer resistance of the NCP-600k is much lower than those of the other NCP electrodes, so do the R_s_ and R_f_. It is demonstrated that the highly dense NCP-600k electrode has higher electrical conductivity and shorter ion transport pathway, which account for its excellent rate performance and cycling capability.

The structural integrity of the electrodes, especially its stability during long-term cycling, is a key ensuring the stability of capacity of LIBs. Figure S4 shows the structure and morphology of the NCP-600k after 500 cycles at 8.0 A g^−1^, which are similar to those before cycling, implying the excellent morphological and structural stability of the NCP-600k. The only recognizable change is the surface of the carbon fiber which turns to be rougher after cycling. Moreover, there are some particles attached on the fiber surface after cycling (Figure S4c). XRD analysis (Figure S5) indicated that the compositions of these particles include C, Li_2_CO_3_, LiF^37^. The HRTEM image and the corresponding SAED pattern (Figure S4d) of a typical carbon fiber from a cycled NCP-600k show a similar amorphous structure as the sample before cycling (Fig. 2e,f). The structural stability of the NCP-600k electrode during long-term cycling is confirmed.

## Conclusion

In summary, a free-standing NCP-600k with highly dense 3D hierarchical porous architecture and excellent bending flexibility is synthesized by pressurized pyrolysis of MF. When used as the anode materials of LIB, the NCP-600k exhibits initial reversible capacity of 480 mA h g^−1^ at 0.05 A g^−1^ and a stable capacity of 329.8 mA h g^−1^ at a current density of 0.5 A g^−1^ after 200 cycles. Even at a high current density of 8.0 A g^−1^, a steady capacity of 126.5 mA h g^−1^ is maintained after 500 cycles, which is superior compared to the performance of the counterpart prepared without compression. Such outstanding electrochemical performance of NCP-600k can be ascribed to (a) the 3D highly dense interconnected carbon network with numerous junctions which can facilitate the efficient electron transfer and provide short transportation paths for lithium ions; and (b) the excellent mechanical flexibility and self-standing capability which exempt the use of binder, conductive additive and current collector. Compared with the existing carbon-based electrode, such as the CNT or graphene-based papers, our NCP shows not only better electrochemical performance but also many practical advantages. For example, the manufacturing method is more economical and easier for scaling up. Moreover, even at a mass loading of 3.2 mg cm^−2^, our NCP still can maintain good flexibility and rate performance, implying a great promise of application in the high-performance energy supplies for the flexible and wearable electronics.

## Methods

### Preparation of the NCP

The NCP is prepared simply by pyrolyzing pressurized melamine foam. Typically, a piece of melamine foam (SINOYQX, Sichuan Province, China) is sliced into blocks with dimensions of 200 mm (L) × 50 mm (W) × 20 mm (H) and then cleaned with acetone and distilled water, followed by overnight drying in vacuum oven at 80 °C. Then, the MF sample is encapsulated into a quartz tube (diameter = 80 mm, length = 1000 mm) with some graphite plates placed on it to provide constant compression. Under the protection of argon gas atmosphere, the MF in the quartz tube is heated from room temperature to 1000 °C at a rate of 5 °C min^−1^ and then kept at 1000 °C for 1 hour. After cooling down to room temperature, a piece of NCP with dimensions around 150 mm (L) × 25 mm (W) is obtained.

### Microscopic characterizations

The morphologies and microstructures of the NCPs before and after electrochemical cycling tests are characterized with scanning electron microscope (Vega 3, TESCAN; JSM-6335F, JEOL) and transmission electron microscope (JEM-2100, JEOL). X-ray diffraction system (D5000, Siemens) and Raman spectroscopy (LabRam HR-800, Horiba) are performed to detect the possible crystalline structure in the NCPs. The elemental composition and chemical state of the NCPs are examined by X-ray photoelectron spectroscopy (ESCaLAB 250, Thermo Scientific). The N_2_ adsorption-desorption isotherms are determined by the Brunauer-Emmett-Teller (BET) measurements conducted with a Micromeritics apparatus (ASAP 2020, Micromeritics Instrument Corporation) at −197 °C, and the pore size distribution (PSD) is acquired from the N_2_ adsorption data based on the Barrett-Joyner-Halenda (BJH) model.

### Test of the critical curvature at fracture under bending

To quantify the bending flexibility of NCPs, the maximum bearable curvature before the fracture is measured by 3-point bending tests using a customized clamp kit on a Microforce Testing System (Tytron 250, MTS), as shown in Fig. 4a. The as-prepared NCPs are cut into rectangular strips with dimensions of 1.0 cm × 4.0 cm. Then, the strip sample is mounted onto the fixed supporting pins. The displacement-controlled load is applied by pushing the third moving pin at the middle point of the sample with a constant rate of 2 mm min^−1^. The force experienced by the loading pin as well as its displacement is recorded until the fracture of the sample happened. According to the Bernoulli-Euler beam theory (see Supplementary Information for details)^38^, the maximum bearable curvature before fracture,${\kappa }_{c}$, and the flexural modulus, ${E}_{{\rm{f}}}$, are given by ${\kappa }_{c}=12{W}_{\max }^{c}/{L}^{2}$ and ${E}_{{\rm{f}}}={L}^{3}S/4a{h}^{3}$ respectively, where *L* is the span between two supporting pins, ${W}_{\max }^{c}$ stands for the deflection of the sample at the middle point at the fracture moment, *a* and *h* are the width and thickness of the sample’s cross section respectively, and *S* is the slope of the initial portion of the force-deflection curve.

### Electrochemical performance measurements

The electrochemical performance of the NCPs is characterized using CR2025-type coin cells. Typically, the as-prepared NCP samples are cut into circular disks with a diameter of 13 mm by mechanical punching and used as the free-standing anode in coin cell LIBs, in which no current collector, binder or conductive additive is applied. A piece of pure lithium foil is used as both the counter electrode and the reference electrode. 1 M LiPF_6_ dissolved in ethylene carbonate (EC)/dimethyl carbonate (DEC)/dimethyl carbonate (EMC) with a volumetric ratio of 1:1:1 is adopted as the electrolyte. And the polypropylene (PP) microporous membrane (Celgard 2300, thickness = 25 μm) is used as the separator. The cells are assembled and sealed in a glove box filled with argon gas. The galvanostatic charge-discharge measurements are performed on a battery testing system (LAND-CT2001A, Wuhan Jinnuo, China) under different current densities with voltage ranging from 0.01 to 3.0 V (vs. Li^+^/Li). Electrochemical impedance spectroscopy (EIS) is performed on an electrochemistry workstation (VMP3, Bio-Logic) by applying AC with voltage amplitude of 5 mV and frequency ranging from 100 kHz to 0.01 Hz. The capacity shown in this study is the values normalized by the mass of the corresponding NCP anode.

## Electronic supplementary material

Supplementary information
