# Supplementary material for: Nitrogen-doped carbon paper with 3D porous structure as a flexible free-standing anode for lithium-ion batteries
Source: Sci Rep. 2017 Aug 10;7:7769. doi: 10.1038/s41598-017-07345-y (PMC5552696; doi:10.1038/s41598-017-07345-y)
Supplement: Supplementary file 1 — Supplementary information [file 41598_2017_7345_MOESM1_ESM.pdf]

Supplementary Information

Nitrogen-doped carbon paper with 3D porous structure as a flexible free-standing anode for lithium-ion batteries

Hua Zhang[1,2], Juntan Yang[2], Haoqing Hou[1], Shuiliang Chen[1*], and Haimin Yao[2*]

[1]Department of Chemistry and Chemical Engineering, Jiangxi Normal University, Nanchang 330022, China
[2]Department of Mechanical Engineering, The Hong Kong Polytechnic University, Hung Hom, Kowloon, Hong Kong SAR, China

[*]Correspondence and requests for materials should be addressed to H.Y. (E-mail: mmhyao@polyu.edu.hk, Tel.: +852 2766 7817) and S.C. (E-mail: slchenjxnu@jxnu.edu.cn, Tel.: +86 791-88120740)

1. Calculation of the density of and porosity of NCPs

The density of the porous NCPs, ρ_{NCP}, is defined as

$$\rho_{\mathrm{NCP}} = \frac{m}{V_{\mathrm{nominal}}} \tag{1}$$

where m and V_{nominal} are the mass and nominal volume of the NCP respectively. For a cuboid NCP sample, the nominal volume, V_{nominal}, is equal to the product of its length, width and height, which can be measured from SEM images. On the other hand, the porosity of a porous material is defined as

$$\phi = 1 - \frac{V_{\mathrm{solid}}}{V_{\mathrm{nominal}}} \tag{2}$$

where V_{solid} stands for the volume the solid material. For a NCP sample, $V_{\mathrm{solid}} = \frac{m}{\rho_{\mathrm{Carbon}}}$, where ρ_{Carbon} is the density of the solid amorphous carbon. Therefore Eq. (2) can be rewritten as

$$\phi = 1 - \frac{V_{\mathrm{solid}}}{V_{\mathrm{nominal}}} = 1 - \frac{m}{V_{\mathrm{nominal}}\rho_{\mathrm{Carbon}}} = 1 - \frac{\rho_{\mathrm{NCP}}}{\rho_{\mathrm{Carbon}}} \tag{3}$$

By taking $\rho_{\text{Carbon}} = 1.8\,\text{g/cm}^3$, the density and porosity for each NCP sample can be calculated from its mass and dimensions (see Table 1) by using Eqs. (1) and (3). The results are summarized in Table 1.

2. Calculation of the maximum bearable curvature before fracture

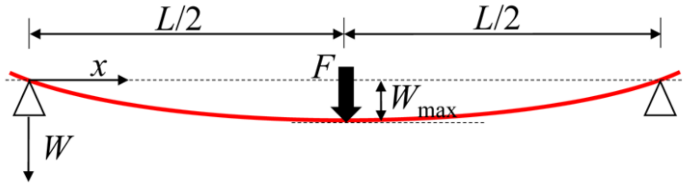

Figure S1. The model diagram of bending test.

A prismatic beam is supported at both ends and subjected to a concentrated force load F at the middle point, as shown in Figure S1. Due to the symmetry, we only consider the left half part of the beam ($0 \le x \le L/2$). According to the Bernoulli-Euler beam theory [1], the deflection of this beam is given by

$$W(x) = Fx\left(3L^2 - 4x^2\right)/48EI \quad (0 \le x \le L/2) \tag{4}$$

where E is the Young's modulus, I refers to the moment of inertia of the beam's cross-section, L presents the span between two supports. The maximum deflection happens at the middle point $(x = L/2)$ and is given by

$$W_{\max} = FL^3/48EI \tag{5}$$

The curvature of the deformed beam can be calculated from the deflection function $W(x)$ through

$$\kappa(x) = -W''/\left(1 + W'^2\right)^{3/2} \tag{6}$$

where W' and W'' are the first and second derivatives of $W(x)$ with respect to x, respectively. It can be easily demonstrated that W'^2 monotonically decreases while W'' monotonically increases as x increases in the range of $0 \le x \le L/2$. Therefore, the maximum curvature, $\kappa_{\max}$, occurs at the middle point $x = L/2$, where $W' = 0$. Inserting $W' = 0$ and $x = L/2$ into Eq. (6), we have $\kappa_{\max} = FL/4EI = 12W_{\max}/L^2$. In

our 3-point bending experiment, the NCP beam was loaded until fracture. The maximum bearable curvature of the NCP, κ_c, is deduced through $\kappa_c = 12W_{\max}^{c} / L^2$ with $W_{\max}^{c}$ being the maximum deflection at the fracture moment.

3. Microscopic characterizations of NCPs

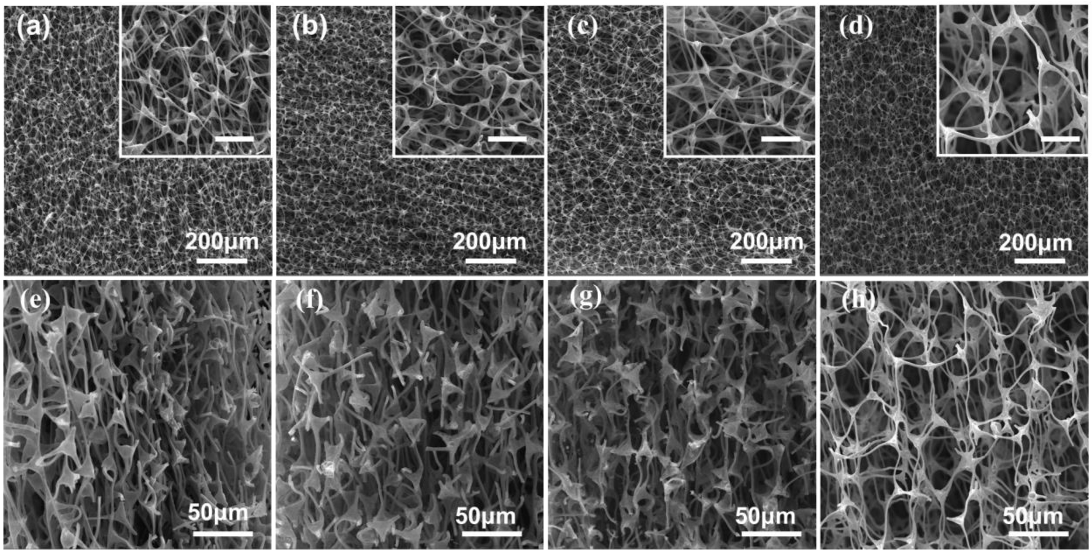

Figure S2. (a-d) SEM images of the surfaces of NCP-600k, NCP-400k, NCP-200k and NCP-0, respectively with insets being the corresponding close-ups. Scale bar = 50 µm. (e-f) Cross-sectional SEM images of NCP-600k, NCP-400k, NCP-200k and NCP-0, respectively.

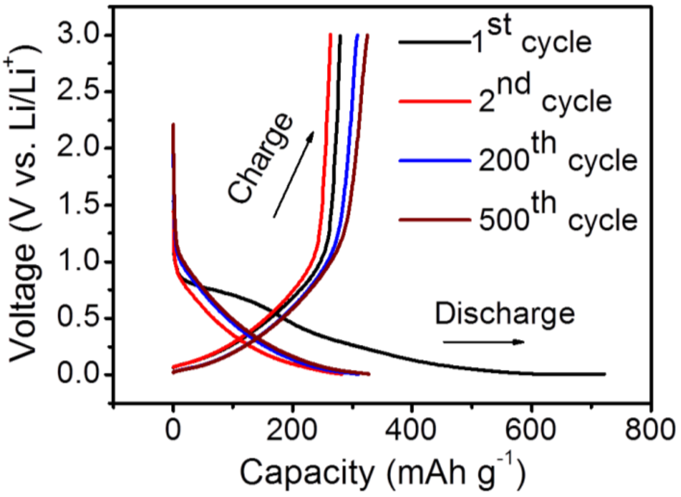

Figure S3. Galvanostatic charge-discharge voltage profiles of the NCP-0 electrode at a current density of 0.05 A g^{-1}.

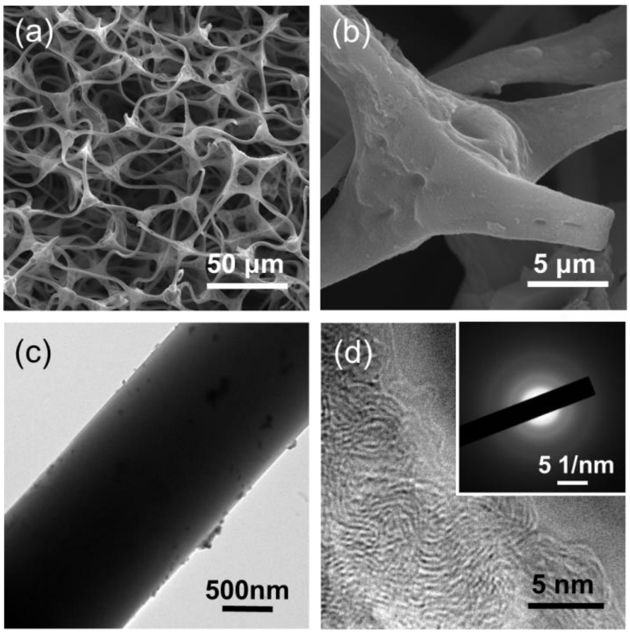

Figure S4. Ultrastructure of the NCP-600k electrode after cycling test (500 cycles). (a) and (b) SEM images; (c) TEM image of a fiber, and (d) HRTEM image of a fiber with inset being the SAED pattern.

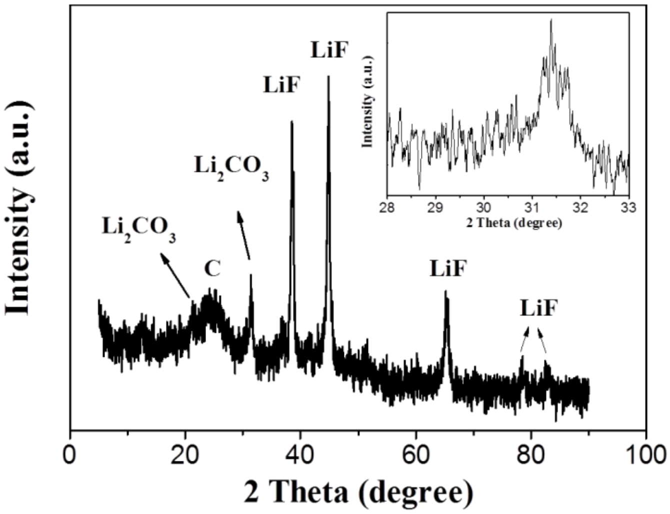

Figure S5. XRD pattern of the NCP-600k electrode after 500 cycles at 8 A g^{-1} with inset being a close-up. The calculated d-spacings are in good consistence with the standard values of C, Li_2CO_3 and LiF from JCPDS files (C: 25-0284; Li_2CO_3: 80-1307; LiF: 02-1111).

Table S1. Comparison of synthesis methods, electrochemical performance of different kinds of flexible carbon-based electrode material

Flexible materials	Synthesis methods	Rate performance	Cycling performance	Ref.
**Graphene film**	Chemical vapor deposition	352 mA h g^{-1} at 0.15 A g^{-1} and 140 mA h g^{-1} at 0.5 A g^{-1}	A capacity of 350 mA h g^{-1} after 50 cycles at 0.15 A g^{-1}	2
**Graphene paper**	Freeze-drying, thermal reduction, and mechanical pressing	557, 268, 169, and 141 mA h g^{-1} at 0.2, 0.5, 1, and 1.5 A g^{-1}	A capacity of 568 mA h g^{-1} after 50 cycles at 0.1 A g^{-1}	3
**Graphene paper**	Vacuum filtration and thermal reduction	150 mA h g^{-1} at 5 A g^{-1} and 70 mA h g^{-1} at 10 A g^{-1}	No apparent capacity loss up to 1000 cycles at 5 and 10 A g^{-1}	4
**Graphene paper**	Vacuum filtration and photoflash/laser-reduction	156 mA h g^{-1} at 14.8 A g^{-1} and 80 mA h g^{-1} at 37.2 A g^{-1}	A stable capacity of 150 mA h g^{-1} after 1000 cycles at 14.8 A g^{-1}	5
**Graphene paper**	Chemical vapor deposition and Vacuum filtration	1350 mA h g^{-1} at 0.05 A g^{-1} and 120 mA h g^{-1} at 1 A g^{-1}	A capacity of 1220 mA h g^{-1} after 100 cycles at 0.05 A g^{-1}	6
**Carbon nanofibers**	Electrospinning and calcination	200, 180, and 152.7 mA h g^{-1} at 0.2, 0.5, and 1 A g^{-1}	A capacity of 460 mA h g^{-1} after 100 cycles at 0.05 A g^{-1}	7
**Carbon nanotube film/ PP separator**	Vacuum filtration	332 mA h g^{-1} at 0.1 A g^{-1} and 80 mA h g^{-1} at 1.2 A g^{-1}	210 mA h g^{-1} after 100 cycles at 0.1 A g^{-1}	8
**Graphite/Carbon nanotube films**	Chemical vapor deposition and cross-stacking	335 mA h g^{-1} at 0.036 A g^{-1} and 326 mA h g^{-1} at 0.72 A g^{-1}	A capacity retention of 99.1% after 50 cycles at 0.036 A g^{-1}	9
**Carbon nanotube films**	Vacuum filtration	175 mA h g^{-1} at 3 A g^{-1} for MWCNT	300 mA h g^{-1} after 40 cycles at 0.025 A g^{-1} for MWCNT	10
**Carbon nanotubes / graphene paper**	Chemical vapor deposition	265 and 55 mA h g^{-1} at 0.06 A g^{-1} and 3.6 A g^{-1}	A stable capacity of 290 mA h g^{-1} at 0.03 A g^{-1} after 40 cycles	11
**Nitrogen-doped Carbon Paper**	**Mechanical stress-pyrolysis**	**428, 320, 240 and 126.8 mA h g^{-1} at 0.1, 0.5, 2 and 8 A g^{-1}**	**A capacity of 329.8 mA h g^{-1} at 0.5 A g^{-1} after 200 cycles and 126.8 mA h g^{-1} at 8 A g^{-1} after 500 cycles**	**present work**

References

1. Gere, J. M. & Timoshenko, S. P. Mechanics of Materials. (PWS Publishing Company, 1997).
2. Rana, K., Kim, S. D. & Ahn, J. H. Additive-free thick graphene film as an anode material for flexible lithium-ion batteries. *Nanoscale* **7**, 7065-7071 (2015).
3. Liu, F., Song, S., Xue, D. & Zhang, H. Folded structured graphene paper for high performance electrode materials. *Adv. Mater.* **24**, 1089-1094 (2012).
4. Zhao, X., Hayner, C. M., Kung, M. C. & Kung, H. H. Flexible Holey Graphene Paper Electrodes with Enhanced Rate Capability for Energy Storage Applications. *ACS Nano* **5**, 8739–8749 (2011).
5. Mukherjee, R., Thomas, A. V., Krishnamurthy, A. & Nikhil, K. Photothermally Reduced Graphene as High-Power Anodes for Lithium-Ion Batteries. *ACS Nano* **6**, 7867–7878 (2012).
6. Ning, G. *et al.* Chemical vapor deposition derived flexible graphene paper and its application as high performance anodes for lithium rechargeable batteries. *J. Mater. Chem. A* **1**, 408-414 (2013).
7. Nan, D. *et al.* Nitrogen-enriched electrospun porous carbon nanofiber networks as high-performance free-standing electrode materials. *J. Mater. Chem. A* **2**, 19678-19684 (2014).
8. Li, X. *et al.* Novel approach toward a binder-free and current collector-free anode configuration: highly flexible nanoporous carbon nanotube electrodes with strong mechanical strength harvesting improved lithium storage. *J. Mater. Chem.* **22**, 18847 (2012).

9. Wang, K. *et al.* Super-Aligned Carbon Nanotube Films as Current Collectors for Lightweight and Flexible Lithium Ion Batteries. *Adv. Funct. Mater.* **23**, 846-853, (2013).

10. Chew, S. Y. *et al.* Flexible free-standing carbon nanotube films for model lithium-ion batteries. *Carbon* **47**, 2976-2983 (2009).

11. Li, S. *et al.* Vertically Aligned Carbon Nanotubes Grown on Graphene Paper as Electrodes in Lithium-Ion Batteries and Dye-Sensitized Solar Cells. *Adv. Energy Mater.* **1**, 486-490 (2011).
